# Case Report: Borrelia-DNA Revealed the Cause of Arthritis and Dermatitis During Treatment With Rituximab

**DOI:** 10.3389/fneur.2021.645298

**Published:** 2021-02-12

**Authors:** Johanna Sjöwall, Georgios Xirotagaros, Chris D. Anderson, Christopher Sjöwall, Charlotte Dahle

**Affiliations:** ^1^Infectious Diseases/Division of Inflammation and Infection, Department of Biomedical and Clinical Sciences, Linköping University, Linköping, Sweden; ^2^Dermatology and Venereology, Department of Biomedical and Clinical Sciences, Linköping University, Linköping, Sweden; ^3^Rheumatology/Division of Inflammation and Infection, Department of Biomedical and Clinical Sciences, Linköping University, Linköping, Sweden; ^4^Clinical Immunology and Transfusion Medicine/Division of Inflammation and Infection, Department of Biomedical and Clinical Sciences, Linköping University, Linköping, Sweden

**Keywords:** Lyme disease, Borrelia-DNA, arthritis, dermatitis, rituximab, Borrelia serology

## Abstract

Borrelia-specific antibodies in serum did not contribute to the diagnosis of Borrelia arthritis or Borrelia-associated dermatitis in a young woman with ongoing treatment with rituximab due to multiple sclerosis. The diagnosis was confirmed by the detection of Borrelia-DNA in a skin punch biopsy. The patient history did not reveal any tick exposure. She had suffered for several months from fluctuating pain and swelling of the right knee as well as skin involvement with redness and oedema around the ankle of the same leg. Monoarthritis was confirmed by a rheumatologist. Knee puncture was performed but the synovial fluid was only sufficient for microscopic examination of crystals. Neither monosodium urate crystals nor calcium pyrophosphate crystals were found. Borrelia serology in blood revealed borderline levels of immunoglobulin (Ig)M and IgG, respectively. Treatment with doxycycline resulted in resolution of the joint and skin manifestations within a month. This case highlights that Borrelia-specific antibody levels cannot be reliably interpreted in patients who have received B-cell depleting therapy. Under these circumstances, detection of the bacterial genome in different body fluids, such as in the skin, can be a useful complement to the diagnosis of Lyme disease. In this young female, the diagnosis would certainly have been further delayed without the detection of Borrelia-DNA in the skin.

## Introduction

An atypical clinical presentation and absence of an adequate immune response to infections are common phenomena in patients with primary immunodeficiency disorders (PID) (1) and multiple sclerosis (MS) (2), as well as in subjects receiving immunosuppressive agents (3). Furthermore, several immune modulating therapies may have similar effects, but this is less well-recognized among physicians lacking deeper knowledge of the impact of the immune system on the pathogenesis and the clinical manifestations of different infectious diseases. As immunomodulating therapies (IMT) are now widely used for a variety of autoimmune and inflammatory diseases, specialists of many different disciplines may need to treat patients using this group of drugs. For instance, patients with MS are often diagnosed and started on treatment at young age and continue for years or decades (4). These circumstances emphasize the importance of physicians being aware of atypical reactions regarding both clinical symptoms and laboratory test results obscuring infections among these patients. This case illustrates that an ongoing infection can easily be overlooked or misinterpreted due to a weak serological response during treatment with a B-cell depleting drug.

## Case Presentation

This case illustrates a 20-year-old female diagnosed with MS at the age of 17. She was initially treated with tocilizumab as *neuromyelitis optica* was suspected due to bilateral optical neuritis and the presence of spinal cord lesions. However, antibodies against aquaporin-4 and myelin oligodendrocyte glycoprotein were not detected and the magnetic resonance imaging (MRI) of the brain and spinal cord as well as cerebrospinal fluid (CSF) findings were supportive of MS. Apart from persistent bilateral severely reduced visual acuity she had no other signs of neurological dysfunction. She had previously been in good health and had no family history of PID, or other systemic inflammatory diseases.

Eighteen months prior to the episode of arthritis and skin symptoms reported here, she was started on off-label treatment with rituximab (RTX). RTX is the most frequently used immunomodulatory drug for MS in Sweden according to the Swedish MS registry (5). Initially, she received 1,000 mg of RTX followed by 500–1,000 mg every 6th month, resulting in depletion of circulating B-cells (<0.001 ×10^9^/L). During this period, there were no signs of neuroinflammatory activity of MS.

### Clinical Episode

**Figure 1:**
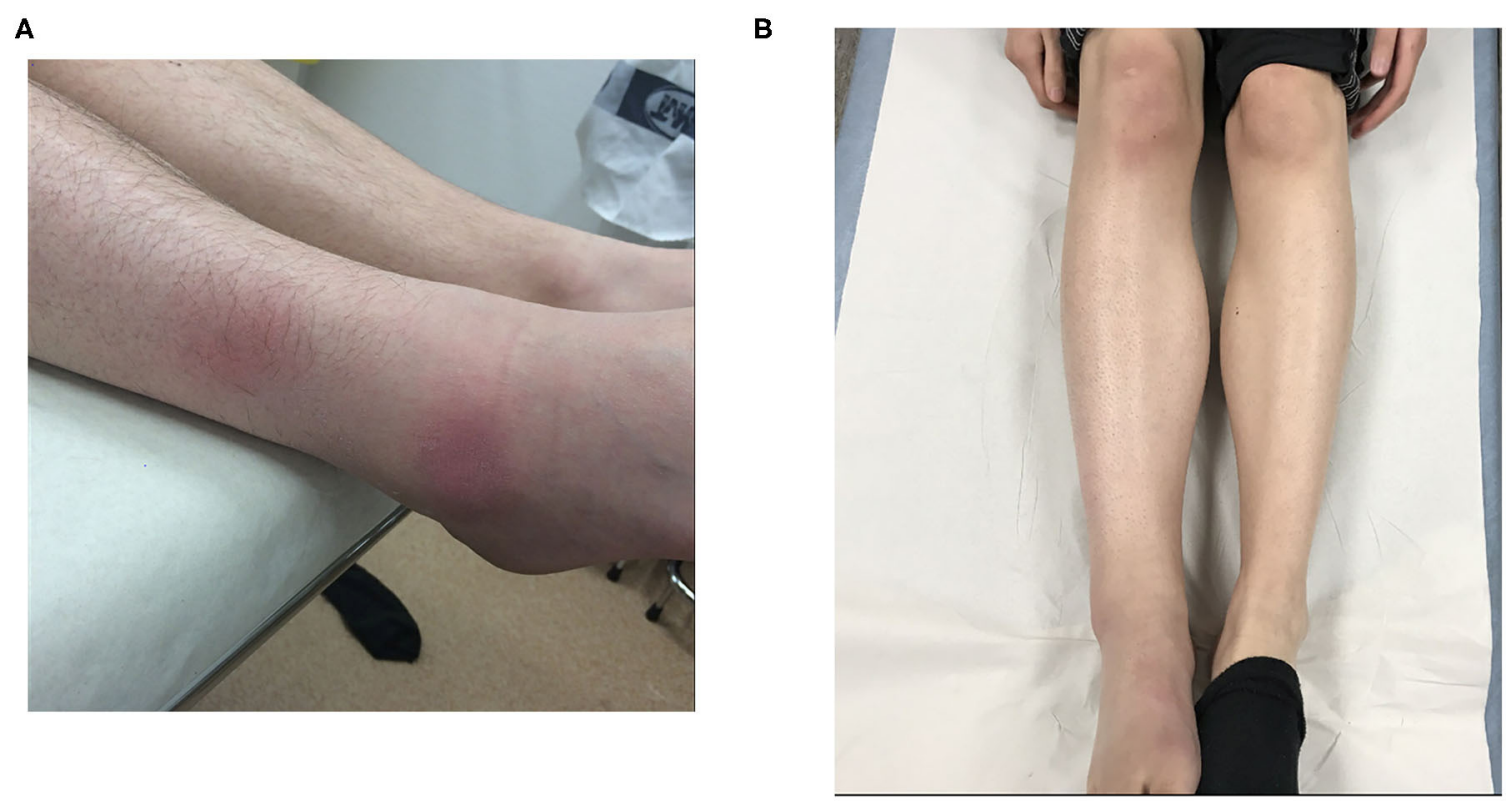
Periarticular swelling of the right leg and ankle. The skin is slightly atrophic adjacent to the two erythematous circular areas seen on the lateral side. The blood vessels appear prominently over the apical part of the foot; a common phenomenon in late cutaneous borreliosis **(A)**. The right knee, calf, and ankle are swollen, without a distinct erythema. Fifteen to twenty degrees deficit in knee extension was observed. Note the dark discoloration of the medial and apical parts of the foot, typically seen in patients with late cutaneous borreliosis **(B)**.

A rheumatologist confirmed the diagnosis of monoarthritis. The right knee had typical signs of inflammation with *rubor, tumor*, and *calor* accompanied by a discretely reduced range of motion. The general status was good without fever. The lower right leg was diffusely swollen and two circular erythematous areas around the ankle were seen (Figures 1A,B). A dermatologist interpreted the skin symptoms as possible panniculitis with atypical erythema nodosum as a potential alternative diagnosis. There were no other clinical or laboratory findings of sarcoidosis.

### Timeline

Treatment with RTX had been ongoing for approximately one and a half year prior to the onset of arthritis and the cutaneous symptoms had been present for at least 6 months prior to the diagnosis. The last dose of RTX was given 1.5 months before the onset of symptoms related to Lyme disease.

### Diagnostic Assessments

Aspiration of synovial fluid resulted in a limited volume, only sufficient for microscopic examination of crystals. Neither monosodium urate crystals nor calcium pyrophosphate crystals were detected in the joint fluid. Duplex ultrasonography of the lower leg showed no signs of deep vein thrombosis and there were no laboratory signs of systemic inflammation. Serological analysis performed 5 months after the last dose of RTX showed borderline levels of immunoglobulin (Ig)M and IgG antibodies against recombinant Borrelia antigens (Liason®, Borrelia IgM detecting OspC and VlsE; Borrelia IgG detecting VlsE). The results were interpreted to be of uncertain clinical significance. Laboratory results are detailed in Table 1.

**Table 1:** Laboratory findings in blood.

**Analyte**	**Results**	**Reference interval**
Hemoglobin	151	117–153 g/L
Leukocyte count	7.2	3.5–8.8 ×10^9^/L
Lymphocyte count	1.3	1.1–4.8 ×10^9^/L
B-cells	<0.001	0.075–0.53 ×10^9^/L
Platelet count	283	160–390 ×10^9^/L
Erythrocyte sedimentation rate (ESR)	2	<21 mm/h
P-C-reactive protein (CRP)	<5	<10 mg/L
S-Creatinine kinase	1.5	<3.6 μkat/L
P-Alanine transaminase	0.35	<0.76 μkat/L
P-Creatinine	81	45–90 μmol/L
P-Urate	209	155–350 μmol/L
S-Angiotensin converting enzyme	0.51	<1.1 μkat/L
Anti-cyclic citrullinated peptide antibody (IgG)	1	<7 U/L
Borrelia antibody (IgM)	38.6	<30 AU/mL
Borrelia antibody (IgG)	16.6	<10 AU/mL

*AU, arbitrary units; P-, analysis in plasma; S-, analysis in serum; U, units*.

Despite the vague antibody results, there were an enduring clinical suspicion of Borrelia infection. Skin biopsies from one of the erythematous areas at the ankle were performed. Standard histopathology showed mild non-specific inflammation. Borrelia-DNA was detected in the biopsy analyzed by polymerase chain reaction (PCR). The method amplifies a 116 base-pair long fragment of the 16S rRNA gene. In addition, a lumbar puncture was done, and CSF was analyzed without presence of intrathecal Borrelia-specific antibodies or elevated levels of the B-cell chemokine CXCL13. Thus, the final diagnosis was Borrelia associated dermatitis and arthritis (Lyme disease).

### Therapeutic Intervention

Prior to the diagnosis of Lyme disease, the patient was prescribed topical steroids for the skin manifestations and the joint symptoms were managed with paracetamol. Once the diagnosis of Lyme disease was confirmed, doxycycline 200 mg once daily for 3 weeks was prescribed. The knee and skin symptoms dissipated during the following month.

### Follow-Up and Outcome

At the last follow-up 1 year after the antibiotic treatment had been ended, there was still minor swelling of the lower leg but no signs of arthritis or dermatitis. An MRI of the lower leg showed mild oedema in musculus soleus and gastrocnemius. Creatinine kinase in plasma was within normal reference.

## Discussion

IMT in general, and particularly B-cell depleting therapies, may be associated with an increased risk of infections (2, 6, 7). Serological screening for IgG against several infectious agents is therefore routinely performed prior to initiation of IMT and vaccination should be considered when immunity is not detected. However, the fact that IMT can have an impact on the clinical picture and serological response to infectious agents is less well recognized among physicians outside the field of immunology and infectious diseases. B-cell depleting therapies are widely used in MS as well as in many other autoimmune diseases, often with a dramatic anti-inflammatory effect and symptom relief (6). In chronic inflammatory diseases like MS, the treatment is often continued for many years and results in undetectable or very low numbers of circulating B-cells. Although RTX, compared to other disease modifying drugs in MS, has been shown to be associated with an increased risk for serious infections it is widely used due its marked effect on the disease activity and disease progression (4). Another side effect is weak and non-protective responses to vaccinations, as long as the circulating B-cells are very low (8, 9).

In the patient described here, Borrelia caused late skin and joint manifestations that did not raise the clinical suspicion of Lyme disease, until the weak serological response was received. Still, the antibody results were interpreted to be of dubious significance. The correct diagnosis was not made until Borrelia-DNA was detected in the skin biopsy. This is consistent with previous and more recent findings of improved diagnostic accuracy using detection of the Borrelia genome in the skin of patients with *acrodermatitis chronica atrophicans*, the late cutaneous manifestation of borreliosis (10, 11). In addition, our case clearly illustrates that, during treatment with B-cell depleting therapies, infections may give rise to an atypical clinical picture as well as a weak serological response to specific pathogens. Awareness of these circumstances should be highlighted to clinicians serving patients on IMT.

### Patient Perspective

The patient had suffered from knee pain and painful skin erythema for several months before the correct diagnosis was identified. She had been in contact with the primary health care several times before the correct diagnosis was made. Despite the fact that she was treated with IMT, her symptoms were initially interpreted as “non-specific findings of uncertain origin.”

## Data Availability Statement

The raw data supporting the conclusions of this article will be made available by the authors, without undue reservation.

## Ethics Statement

Ethical review and approval was not required for the study on human participants in accordance with the local legislation and institutional requirements. The patient provided her written informed consent to participate in this study. Written informed consent was obtained from the individual(s) for the publication of any potentially identifiable image or data included in this article.

## Author Contributions

JS, CS, and CD had full access to all of the data and takes responsibility for the integrity, accuracy and interpretation of the data. All authors contributed to the article and approved the submitted version. All authors were involved in drafting the manuscript or revising it critically for important intellectual content and all authors approved the final version to be published.

## Conflict of Interest

The authors declare that the research was conducted in the absence of any commercial or financial relationships that could be construed as a potential conflict of interest.
